# Safety and factors affecting diagnostic yield in modified virtual bronchoscopy-guided transparenchymal nodule access for pulmonary lesions

**DOI:** 10.1007/s00464-025-11865-4

**Published:** 2025-06-05

**Authors:** Yuan-Ming Tsai, Ung-Kai Ting, Ying-Yi Chen, Kuan-Hsun Lin, Hsu-Kai Huang, Tsai-Wang Huang, Hsueh-Liang Hsu

**Affiliations:** 1 Division of Thoracic Surgery, Department of Surgery, Tri-Service General Hospital, National Defense Medical Center, Taipei City, Taiwan; 2 School of Medicine, National Defense Medical Center, Taipei City, Taiwan; 3 Taipei Veterans General Hospital Taoyuan Branch, Taoyuan City, Taiwan

**Keywords:** Virtual bronchoscopic navigation, Transparenchymal nodule access, Image guidance, Pulmonary lesions, Diagnostic yield, Safety

## Abstract

**Background:**

With the advent of low-dose computed tomography (CT) screening, minimally invasive evaluation of pulmonary lesions has become increasingly important. This study aimed to assess factors influencing the diagnostic yield of novel bronchoscopic transparenchymal nodule access (BTPNA) method for diagnosing pulmonary lesions in a real-world clinical setting.

**Methods:**

This retrospective study evaluated patients who underwent BTPNA with or without real-time image guidance for pulmonary lesion assessment. Lesions were deemed inaccessible if a diagnosis could not be obtained via CT-guided transthoracic biopsy or conventional bronchoscopy. CT images for each patient were used to reconstruct a navigation map and design a point of entry (POE) along the airway wall through the lung parenchyma to the lesion. Patients were monitored for more than 48 h after BTPNA.

**Results:**

Between January 2022 and July 2024, 17 patients underwent BTPNA without adverse events requiring immediate treatment during the procedures. Adequate biopsies were obtained from 15 patients for whom a tunnel path was created. The overall diagnostic yield was 77%, influenced by nodule size, CT morphology, and the chosen path. Real-time guidance during BTPNA and airway diameter at the POE did not significantly affect the diagnostic yield in this analysis.

**Conclusion:**

BTPNA has shown potential as a high-yield and safe diagnostic technique for patients in whom alternative methods were considered unsuitable due to safety concerns or low expected diagnostic yield.

**Supplementary Information:**

The online version contains supplementary material available at 10.1007/s00464-025-11865-4.

Lung cancer remains the leading cause of cancer-related deaths worldwide. Studies such as the NELSON, NLST, and UKLS trials have shown that chest computed tomography (CT) screening reduces lung cancer mortality by 20% [1]. As lung cancer screening programs become more widespread, the number of pulmonary lesions requiring evaluation is expected to increase, underscoring the need for advancements in diagnostic and therapeutic tools [2]. Transthoracic needle aspiration (TTNA) and conventional bronchoscopy have traditionally been the preferred methods for sampling pulmonary lesions. Although transthoracic procedures are highly accurate for lesions > 20 mm, they carry a significant risk of complications, including pneumothorax rates of up to 26% and chest drain insertion in 6.9% of cases [3]. The pooled diagnostic yield is 75% for the bronchoscopic approach and 93% for the transthoracic approach, with the latter being superior for lesions ≤ 2 cm (92% vs. 66%) [4]. Alternatives to surgical diagnosis for screening-detected nodules, with or without localization, can undermine the value of screening due to the cost implications and risks associated with surgical procedures [5]. Additionally, the invasiveness of surgical resection and risk of conversion to thoracotomy are significant concerns. Surgical approaches are particularly unsuitable for centrally located pulmonary lesions, which typically require anatomical resections such as lobectomy or segmentectomy.

To enhance diagnostic yield and safety, advanced techniques such as ultrathin bronchoscopy, radial-probe endobronchial ultrasound (EBUS), and electromagnetic navigation bronchoscopy have been developed. These methods are often combined with fluoroscopy or cone-beam CT for positional verification, achieving diagnostic yields of approximately 70% with a low pneumothorax rate of 2% [6]. However, the effectiveness of these techniques can vary based on factors such as nodule size, proximity to the hilum, and the presence of a “bronchus sign” on CT scans [7]. If no visible bronchus leads to the lesion, navigation through multiple subsegments becomes challenging. A novel approach involves using virtual bronchoscopy navigation (VBN) to access lesions via a transparenchymal route, demonstrating a sensitivity of approximately 77% (range 72–82%) for obtaining a histopathological diagnosis [6]. Unlike the CT-guided transthoracic approach, bronchoscopic transparenchymal nodule access (BTPNA) may be particularly advantageous for nodules in specific anatomical locations, such as centrally located lesions or those difficult to reach via the transthoracic route. Nonetheless, further comprehensive data are needed to better understand the correlation between diagnostic yield and specific influencing factors as well as the real-world applicability of these approaches to pulmonary lesions [8].

This study aimed to evaluate the feasibility and identify factors influencing the diagnostic accuracy of the BTPNA procedure, specifically addressing the current lack of data on its application without the use of the full sheath and balloon set, as well as its performance in real-world clinical settings. Additionally, we sought to describe this modified technique and summarize its efficacy and safety.

## Materials and methods

This single-center retrospective cohort study included patients who underwent the BTPNA procedure for the assessment of pulmonary lesions. Patients were recruited between January 2022 and July 2024. The inclusion criteria were as follows: age ≥ 18 years, pulmonary lesions suspected to be malignant or metastatic from an unknown primary tumor, lesions unlikely to yield a diagnosis via conventional transbronchial approach due to the peripheral location and absence of a bronchus sign, lesions anatomically shielded by the scapula, ribs or adjacent to the diaphragm, and centrally located lesions associated with a high risk of complications, but considered accessible via VBN. Exclusion criteria included any contraindications to bronchoscopy, such as inability to discontinue anticoagulant or antiplatelet therapy near the procedure, intolerance to general anesthesia, pregnancy or breastfeeding, moderate to severe pulmonary fibrosis, and severe emphysema with bullae > 5 cm along the planned tunnel route to the target lesion. This study was approved by the institutional ethics committee (No. C202205053), and the requirement for informed consent was waived.

### Planning stage and procedure

**Fig. 1:**
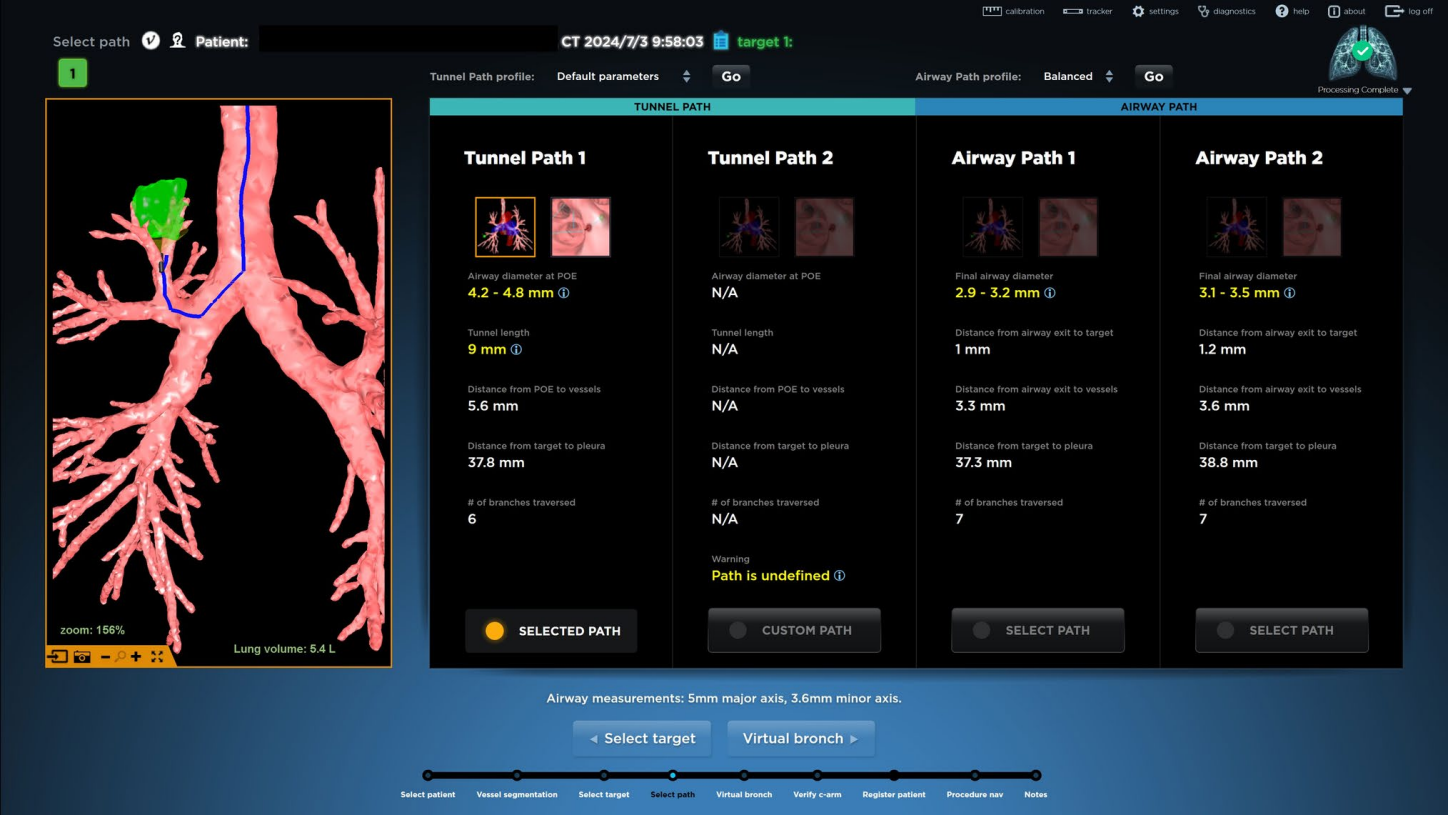
Visualization and characteristics of planning route (tunnel or airway) using the Archimedes System (Broncus Medical, Inc.)

**Fig. 2:**
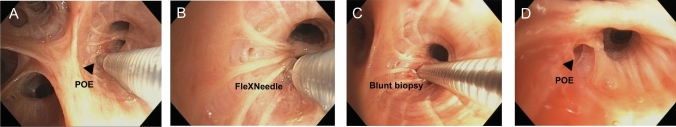
Creation of a transparenchymal tunnel for nodule access under virtual guidance. **A** The bronchoscope is guided to the point of entry (POE) using virtual navigation. **B** A coring needle (FleXNeedle; Broncus Medical) is inserted through the bronchoscope’s working channel to create the initial entrance point at the POE. **C** Blunt biopsy forceps are employed to extend the pathway and create a tunnel through the lung parenchyma toward the lesion. **D** The completed tunnel allows access to the lesion for sample collection, enabling precise targeting for diagnosis without bleeding

The Archimedes VBN system (Broncus Medical, Inc., San Jose, California, USA) was used to reconstruct chest CT data (1-mm slices) into a three-dimensional model of the bronchial airways, ribs, lungs, and vascular structures [9, 10]. The system generated an airway route and identified the point of entry (POE) on the airway wall, providing a direct, vessel-free path (the tunnel path) through the lung parenchyma to the pulmonary lesion (Fig. 1). The bronchoscopic procedure was performed under general anesthesia with muscle paralysis, either in a hybrid operating room or a conventional operating room. All procedures were performed by Y.M.T., a thoracic surgeon with extensive experience in advanced bronchoscopy, including over 100 VBN procedures and prior training in BTPNA through simulation, animal models, and participation in an earlier feasibility study [10, 11]. Upon reaching the POE under virtual guidance, an 18 gauge coring needle (FleXNeedle; Broncus Medical) with an outer diameter (OD) of 1.9 mm (mm) and an adjustable penetration depth ranging from 0 to 20 mm in 5 mm increments, was inserted through a bronchoscope, either the BF-P60 (2.2 mm channel width, 4.9 mm OD) or the BF-H190 (2.0 mm channel width, 5.5 mm OD) (Olympus Medical Systems Corp., Tokyo, Japan) to create an entrance point. Olympus flexible round cup biopsy forceps (FB-19K-1; compatible with 2.0 mm channel) were then used to create a tunnel through the lung parenchyma, following the preprocedural plan, to access the target lesion for tissue sampling (Fig. 2). In the hybrid operating room, cone-beam CT was used to confirm the biopsy location, whereas in the conventional operating room, a C-arm X-ray instrument was utilized. Throughout the procedure, biopsies or cytology samples were obtained whenever possible. On average, two to three biopsy passes were performed per lesion. Mediastinal staging with EBUS was not routinely performed in conjunction with BTPNA procedure, but was typically conducted separately, based on multidisciplinary team decisions and guided by radiographic findings or confirmed pathological diagnoses. A chest X-ray was performed 4 h after the BTPNA procedure to monitor complications, which were recorded if present. Surgical resection was performed after a mean interval of 14 days.

### Outcomes

Diagnostic yield was defined as instances where the biopsy results matched the final diagnosis confirmed by pathological and/or bacterial assessment of the bronchoscopic samples. Procedure time was measured from the start of the navigation procedure until the completion of the entire BTPNA and anesthesia. Safety was assessed by monitoring for intraprocedural complications, such as localized bleeding or cardiopulmonary instability, as well as postprocedural events, including pneumothorax and hemothorax. Additional parameters evaluated included the type of guidance used during the navigation procedure and preprocedural evaluations via the Archimedes VBN system, such as lung volume, airway diameter at the POE, tunnel length, POE-to-vessel distance, and target-to-pleura distance.

### Statistical analysis

Categorical variables were summarized as frequencies and percentages, while continuous variables were described using mean, minimum, and maximum values. Categorical variables were compared using Pearson’s Chi-squared test or Fisher’s exact test, as appropriate. Continuous variables were compared using the Student’s t-test. Multivariate logistic regression model was used to adjust for potential confounders affecting diagnostic yield, including lesion size, tunnel length, POE-to-vessels distance, lesion-to-pleura distance, and CT morphology. All statistical analyses were performed using SPSS software (version 22; SPSS Inc., Chicago, IL, USA). A two-sided *P*-value of < 0.05 was considered statistically significant.

## Results

From January 2022 to July 2024, 17 patients underwent the BTPNA procedure at our center. The characteristics of the patients and pulmonary lesions are detailed in Table 1. The median age of the patients was 64 years, with the majority being male (70.6%), and the median body mass index was 23.8 (range 19.4–31.6) kg/m^2^. The primary reason for requesting a biopsy was the presence of lesions in patients with a history of malignancy, including prior lung cancer relapse (64.7%). Among all patients, 11.8% were diagnosed with chronic obstructive pulmonary disease. Most lesions were solid (76.5%), predominantly located in the upper lobes, with 47.0% in the right upper lobe and 11.8% in the left upper lobe. The median longest diameter of the nodules was 29.0 (range 8.5–89.1) mm. Among all nodules, 35.3% had a diameter ≤ 20 mm, whereas 64.7% had a diameter > 20 mm.

**Table 1:** Patient and nodule characteristics

Total number of patients	N = 17
Age, median (range; years)	64 (48–81)
Sex, number (%)	
Male	12 (70.6%)
Female	5 (29.4%)
Height, median (range; cm)	164.3 ± 6.6(149–180)
Body weight, median (range; kgw)	64.5 ± 11.9 (43–87)
BMI, median (range)	23.8 ± 3.6 (19.4–31.6)
Smoking, number (%)	7 (41.2%)
Pack Years, median	37.78
Indication for the procedure, number (%)	
with history malignancy, including relapse of prior lung cancer considered	11(64.7%)
without history of malignancy	6 (35.3%)
Chronic Obstruction Pulmonary Disease	2 (11.8%)
CT morphology, number (%)	
Solid	13 (76.5%)
Subsolid	3 (17.6%)
Ground glass opacity	1 (5.9%)
Location SPN, number (%)	
Right upper lobe	8 (47.0%)
Right middle lobe	3 (17.6%)
Right lower lobe	2 (11.8%)
Left upper lobe	2 (11.8%)
Left lower lobe	2 (11.8%)
Lesion longest diameter, median (range; in mm)	29.0 (8.5–89.1)
Lesion per diameter, number (%)	
Diameter ≤ 20 mm	6 (35.3%)
Diameter > 20 mm	11 (64.7%)

*CT* computed tomography

Table 2 provides details of the procedural characteristics and the use of virtual navigation software during the BTPNA procedures. The choice of POE, tunnel path, or a combination thereof was made based on navigational planning and the surgeon’s discretion. The median procedure time was 89.9 (range 40–186) min. The overall diagnostic yield was 76.5%, with 13 of 17 patients successfully diagnosed. The type of guidance used during the VBN procedure varied, with cone-beam CT being the most common (52.9%), followed by no guidance (23.6%), fluoroscopy (17.6%), and mobile C-arm X-ray (5.9%). The patients’ median lung volume was 3.91 L. The median airway diameter at the POE was 5.2 (range 3.2–7.9) mm. The median tunnel length created for access was 17.6 (range 3.0–37.5) mm. Additionally, the median distance from the POE to the nearest vessels was 2.6 (range 0.5–5.6) mm, and the median distance from the target nodule to the pleural surface was 26.4 (range 0.0–57.2) mm. Further descriptive comparison of procedural parameters between diagnostic success and failure groups is summarized in Supplementary Table 1.

**Table 2:** Characteristics of procedural and Archimedes virtual navigation software

Procedure time, median (range; in min)	89.9 (40–186)
Diagnostic yield of VBN procedure (%)	13 (76.5%)
Guidance during VBN procedure, number (%)	
None	4 (23.6%)
Fluoroscopy	3 (17.6%)
Mobile C-arm X-ray	1 (5.9%)
Cone-beam CT	9 (52.9%)
Lung volume (L)	3.91(2.1–6.4)
Airway diameter at the POE (mm)	5.2 (3.2–7.9)
Tunnel length (mm)	17.6 (3.0–37.5)
Distance from POE to vessels (mm)	2.6 (0.5–5.6)
Distance from targets to pleural (mm)	26.4 (0–57.2)

*VBN* virtual bronchoscopic navigation, *POE* point of entry

The independent predictors of diagnostic yield in BTPNA, as identified via multivariate logistic regression analysis, are presented in Table 3. Smaller lesion size was significantly associated with lower diagnostic yield (95% confidence interval [CI], − 3.403 to − 0.631; *P* = 0.011). In contrast, solid CT morphology positively influenced diagnostic yield (95% CI, 0.222 to 1.283; *P* = 0.011). Tunnel length and the distance from the lesion to the pleura were also significant, with longer tunnels (*P* = 0.011) and closer pleural proximity (*P* = 0.035) both associated with reduced yield. Notably, greater distance from the POE to adjacent vessels correlated with improved diagnostic success (*P* = 0.013). However, the use of real-time image guidance during BTPNA was not a statistically significant factor (*P* = 0.112), suggesting that procedural planning, anatomical factors, and vessel-free trajectories may facilitate more effective sampling.

**Table 3:** Multivariate logistic regression analysis of independent predictors of diagnostic yield

Variable	Unstandardized coefficient (B)	*P*-value	95% Confidence Interval
Lesion per diameter	− 2.017	0.011	− 3.403, − 0.631
Location	− 0.051	0.587	− 0.265, 0.162
CT morphology	0.752	0.011	0.222, 1.283
Guidance during VBN procedure	− 0.214	0.112	− 0.492, 0.065
Lung volume	0.039	0.642	− 0.150, 0.227
Airway diameter at the POE	0.362	0.222	− 0.081, 0.292
Tunnel length	− 0.122	0.011	− 0.206, − 0.038
Distance from POE to vessels	0.355	0.013	0.101, 0.609
Distance from targets to pleural	− 0.031	0.035	− 0.059, − 0.003

*CT* computed tomography, *VBN* virtual bronchoscopic navigation, *POE* point of entry

A biopsy was successfully performed in 15 patients in whom the BTPNA procedure was completed, yielding a procedural success rate of 88.2% (Table 4). In two patients (cases 11 and 16), the procedure was aborted due to localized bleeding at the POE, which led to early termination of the procedure without biopsy. No major complications, such as pneumothorax or hemothorax requiring chest tube placement or significant respiratory compromise were observed. All patients were monitored for at least 48 h post-procedure, and chest X-rays confirmed the absence of pneumothorax requiring intervention. In all malignancy cases, the collected tissue was adequate for molecular testing to support treatment decisions. For cases initially identified as malignant, such as poorly differentiated carcinoma and adenocarcinoma in patients 4, 6, 14, and 17, the final diagnoses confirmed through thoracoscopic resection aligned with these malignant findings. Similarly, benign lesions, such as chronic inflammation or fibrosis, were initially suggested in patient 8 and later confirmed, demonstrating the high diagnostic accuracy of surgical resection in providing definitive diagnoses. Importantly, pathological analysis revealed minimal inflammation or sparse degenerative atypical cells in some cases. However, further surgical evaluation led to a final diagnosis of adenocarcinoma in patients 1 and 13 and necrotizing inflammation with abscess formation in patient 5. These findings underscore that less-invasive initial findings may sometimes underrepresent the severity of underlying pathology (Table 4).

**Table 4:** Per case data

	Age	Gender	Segment	Size (mm)	Guidance	Procedure time	Tunnel length (mm)	Lesion to pleural (mm)	Pathology	Further diagnostic method	Final result
1	56	female	LB4	27	Fluoroscopy	89	3	24.7	Minimal inflammation with scant atypical cells	VATS wedge resection	AdenoCa
2	81	male	RB4	40	Fluoroscopy	121	4	57.2	Poorly differentiated carcinoma		
3	50	male	RB7	23.9	Cone-beam CT	107	17	0	Malignant solitary fibrous tumor		
4	69	male	RB2	32	None	55	4	33.9	Poorly differentiated carcinoma	VATS lobectomy	AdenoCa
5	78	male	RB1	21	Cone-beam CT	56	20	10.7	Scant degenerative atypical cells present	VATS lobectomy	Necrotizing inflammation with abscess formation
6	48	male	RB4	30	None	40	5	43.6	AdenoCa	VATS lobectomy	AdenoCa
7	64	female	RB9	14	Cone-beam CT	124	28	34.4	Suspicious of malignancy	VATS lobectomy	Atypical carcinoid tumor
8	62	female	RB3	38	None	75	13	10.2	Chronic inflammation with fibrosis	VATS wedge resection	Organization changes with chronic fibrosis
9	71	male	RB4	19	Cone-beam CT	96	3.3	34.9	Chronic inflammation		
10	49	female	LB7 + 8	12	None	116	16.5	42.7	Chronic inflammation		
11	70	male	RB1	17	Cone-beam CT	64	26	38.3	Bronchial epithelial fragments, focal dust deposits (bleeding)	VATS lobectomy	AdenoCa
12	59	male	LB10	8.5	Cone-beam CT	49	25.5	21.3	Atypical adenomatous hyperplasia		
13	63	male	RB1	40.5	Cone-beam CT	100	22	37.8	Chronic inflammation with atypical adenomatous hyperplasia	VATS lobectomy	AdenoCa
14	49	female	LB1 + 2	32.5	C-arm	74	16	9.2	Non-small cell carcinoma	VATS lobectomy	AdenoCa
15	70	male	RB1	20.2	Fluoroscopy	66	37.5	12.1	Mild chronic inflammation		
16	78	male	RB1	89.1	Cone-beam CT	110	28.5	0	Benign bronchial epithelium with fibrosis(bleeding)		
17	71	male	RB1	28.3	Cone-beam CT	186	9	37.8	AdenoCa	RATS lobectomy	AdenoCa

## Discussion

This monocentric study conducted in a real-world setting evaluated transparenchymal lesion access via BTPNA with or without image guidance and assessed the contribution of vessel mapping technology to the safe and effective sampling of pulmonary lesions without a bronchus sign. This study represents the first evaluation of a modified BTPNA (mBTPNA) technique, performed without the standard Archimedes sheath (OD 2.65 mm) and dilated balloon set (OD 1.9 mm), which are typically used to facilitate tunnel creation. A flexed needle was utilized to create the POE according to the design generated using the Archimedes system, after which the procedure relied solely on flexible biopsy forceps to create the transparenchymal path with and without real-time imaging. Our findings demonstrated a diagnostic yield of 76.5%, consistent with previously reported results [6, 9, 12]. Excluding the two patients in whom a successful tunnel path was not achieved, the diagnostic yield increased to 86.7% (13/15). The mBTPNA was performed with minimal adverse events and no cases of pneumothorax or respiratory failure.

Our study highlights the nuanced interplay between procedural variables and diagnostic outcomes in BTPNA procedures. Multivariate analysis revealed that smaller lesion sizes were less likely to yield a positive diagnosis. Conversely, favorable CT morphology, characterized by solid components, well-defined margins, and the absence of extensive ground-glass opacity or internal necrosis, was associated with a higher diagnostic yield, emphasizing the importance of nodular radiologic features in enhancing biopsy accuracy. Interestingly, although tunnel length was identified as an independent predictor of diagnostic success, imaging guidance during the procedure did not significantly affect the outcomes. This finding suggests that the complexity of airway navigation can be effectively managed through the use of a dedicated virtual navigation map. Such navigation aids are crucial during the procedure, as they help ensure a safe route and the collection of sufficient samples. This perspective aligns with findings reported in the journals *Thorax* [13] and *Respirology* [14], which indicated that tunnel lengths between 50 and 90 mm are both safe and effective for achieving diagnostic objectives. These results underscore the critical role of meticulous procedural planning and the value of advanced navigation tools in enhancing the success of diagnostic biopsies.

BTPNA technology was developed to overcome the reliance on an airway leading to the lesion. In cases without a bronchus sign, the reported diagnostic yield ranges from 31 to 62% [6, 9]. Thorough preparation, including preprocedural CT imaging and careful route planning, is vital for achieving diagnostic accuracy. However, several challenges, such as poor lesion resolution, airway obstructions, patient positioning discrepancies, and nodule movement during anesthesia, especially for nodules ≤ 2.5 cm located in the lower lobes, can hinder diagnostic outcomes. Advanced imaging techniques, such as cone-beam CT with body-shaped sensing can help mitigate these issues and improve diagnostic accuracy. In our cohort, the distance of the lesion to the pleural surface appeared to influence diagnostic complexity. Additionally, upper-lobe lesions were more prevalent, reflecting findings from lung cancer screening studies [15] and highlighting the difficulty of diagnosing apical nodules using conventional bronchoscopy or CT-guided biopsy. Accessing the lung apex, particularly in the left upper lobe, is limited by the proximity of the aorta and pulmonary arteries, making it challenging to adjust the angulation of the sheath and orient the dissection tip toward the desired direction [16]. Although TTNA remains a standard diagnostic method, its associated risks, such as pneumothorax, are notable. The mBTPNA with forceps may serve as a less invasive alternative to TTNA, especially for nodules located in anatomically challenging regions. By leveraging the bronchoscopic route, mBTPNA reduces the need for percutaneous access and may minimize these complications. This underscores the potential of mBTPNA as a reliable diagnostic tool, particularly in cases where other methods are limited or carry higher risks.

VBN procedures in the upper lobes are challenging due to the difficulty of scope angulation when advancing forceps, brushes, or needles. A randomized trial demonstrated that the diagnostic yield for pulmonary lesions was higher with a 3-mm ultrathin bronchoscope (70.1%) compared to a 4-mm bronchoscope combined with radial EBUS, VBN, and fluoroscopy (58.7%) [17]. Although ultrathin bronchoscopes may improve diagnostic yield, they are limited by the insufficient tissue samples they collect and their dependence on lesions with a visible bronchus sign. Transbronchial needle aspiration (TBNA) and transbronchial biopsy yielded a diagnostic rate of 59.1% and 22.6%, respectively. However, in bronchus sign-negative lesions, the diagnostic yield dropped to 31% even when using electromagnetic navigation bronchoscopy [18]. The absence of a bronchus sign, indicating the lack of direct airway leading to the lesion, is a well-recognized predictor of lower diagnostic outcomes in conventional bronchoscopic approaches. Without a visible airway pathway, accurate navigation and adequate tissue sampling become significantly more challenging. This anatomical limitation often necessitates more invasive techniques such as CT-guided transthoracic biopsy or surgical resection. Moreover, bronchus sign-negative lesions are frequently located in peripheral or apical segments, where access is inherently difficult due to sharp angulation and limited scope maneuverability. These challenges highlight the need for more adaptable bronchoscopic technologies and flexible yet sufficiently robust endoscopic instruments that can facilitate safe and effective tissue acquisition in anatomically unfavorable regions [6, 13].

The biopsy yield for BTPNA was higher than that for guided TBNA (86.3% vs. 67.2%) and demonstrated a diagnostic yield superior to other methods for pulmonary nodule biopsy. In a previous study, a tunnel pathway was successfully created in 83.3% (10/12) of patients, with adequate biopsies correlating with histological findings in all 10 cases [13]. A global multicenter study reported BTPNA biopsy yields of 86.3%, with yields exceeding 90% in lesions where the tunnel length was ≥ 30 mm [14]. The BTPNA procedure was shown to be highly feasible, with successful navigation to targeted nodules in all cases. The diagnostic yield was notably high, with favorable histological results obtained across all cases. These findings underscore the potential of BTPNA as a reliable diagnostic tool, particularly in cases where other methods are limited or carry higher risks.

The decision to use imaging guidance was based on multiple factors, including lesion location, equipment availability, procedural complexity, and patient condition. In this study, cone-beam CT was used selectively to confirm the final position of the biopsy forceps rather than throughout the procedure. Although cone-beam CT was available in our institution, it was not routinely employed due to scheduling constraints and institutional workflow limitations. Notably, the distance from the lesion to the pleura correlated with diagnostic yield, underscoring the challenges of sampling peripheral lesions abutting the pleural surface. Adjunct imaging modalities such as cone-beam CT are increasingly important in improving real-time navigation and confirming tool-to-target alignment, especially for peripheral or anatomically complex pulmonary lesions. These technologies can help compensate for CT-to-body divergence, patient movement, and respiratory variations, which are common challenges during bronchoscopic procedures. While robotic-assisted bronchoscopy combined with cone-beam CT could theoretically improve navigation and sampling, particularly for anatomically challenging lesions [19], our simplified VBN-guided BTPNA approach demonstrated high feasibility and effective transparenchymal access without routine real-time image guidance. Furthermore, our multivariate analysis demonstrated that the type of guidance used was not an independent predictor of diagnostic yield, suggesting that although cone-beam CT may improve procedural visualization, its influence on diagnostic outcomes in BTPNA is likely secondary to factors, such as tunnel length and lesion characteristics.

We observed a notable correlation between the initial pathological findings and the final results, affirmed by subsequent surgical interventions. The few discrepancies underscored the challenges of diagnosing lung lesions solely based on initial biopsies, particularly for small or strategically located lesions. For instance, in patients 1 and 5, initial pathology results showed atypical cells, but further evaluation revealed different diagnoses: adenocarcinoma and inflammation, respectively. These lesions were located in the upper lobes and were characterized by relatively small sizes and ground-glass or mixed attenuation on CT imaging. Such radiologic features and sampling limitations in upper lobe locations may contribute to nondiagnostic outcomes. These cases highlight that initial, less invasive biopsy results may underestimate the severity of the underlying pathology, reinforcing the need for surgical intervention to confirm the nature of suspicious lesions. The nuanced interplay of lesion characteristics and procedural techniques suggests that although smaller lesions pose challenges, biopsy success can be enhanced by optimizing navigation and sampling strategies. The mBTPNA represents a streamlined and adaptable approach for diagnosing pulmonary lesions, capable of being effectively implemented even in settings without advanced image guidance systems, while still yielding favorable histological outcomes.

## Limitations

This study has several limitations. First, it was conducted at a single center with a relatively small sample size, which may limit the generalizability of the findings. Second, the retrospective design may introduce selection bias and limit the ability to control for potential confounding factors. Third, all procedures were performed by a single experienced operator, which ensured procedural consistency but may not reflect the variability in outcomes that could occur among less experienced practitioners. Additionally, the learning curve associated with VBN and BTPNA was not formally assessed. For broader clinical adoption, structured training programs, including simulation-based modules and supervised proctoring, may be necessary to ensure procedural safety and reproducibility during the early phases of implementation.

## Conclusions

Our results demonstrated that the use of VBN to access lesions via the transparenchymal route significantly improved the diagnostic utility of bronchoscopy for traditionally challenging nodules. However, further prospective, multicenter studies with standardized protocols are warranted to comprehensively evaluate the advantages and limitations of the procedure, particularly its use with advanced imaging modalities and sub-centimeter lesions, which are essential for stage-shifting and early lung cancer detection.

## Supplementary Information

Below is the link to the electronic supplementary material.

Supplementary file1 (DOCX 17 KB)
