# Supplementary material for: Safety and factors affecting diagnostic yield in modified virtual bronchoscopy-guided transparenchymal nodule access for pulmonary lesions
Source: Surg Endosc. 2025 Jun 5;39(7):4662–70. doi: 10.1007/s00464-025-11865-4 (PMC12222317; doi:10.1007/s00464-025-11865-4)
Supplement: Supplementary file 1 — Supplementary file1 (DOCX 17 KB) [file 464_2025_11865_MOESM1_ESM.docx]

| Supplementary Table 1. Procedural parameters by diagnostic outcomes of mBTPNA | | | |
| --- | --- | --- | --- |
|  | Diagnostic success by mBTPNA | | |
|  | Yes (*N* = 13, 76.5%) | No (*N* = 4, 23.5%) | P value |
| Procedure time | 89.6±40.8 | 90.8±19.8 | 0.141 |
| Guidance of mBTPNA |  |  | 0.536 |
| None | 4 (30.8%) | 0 (0.0%) |  |
| Fluoroscopy | 2 (15.4%) | 1 (25.0%) |  |
| Mobile C-arm X-ray | 1 (7.7%) | 0 (0.0%) |  |
| Cone-beam CT | 6 (46.1%) | 3 (75.0%) |  |
| Lung volume (L) | 3.8±1.1 | 4.2±1.6 | 0.586 |
| Airway diameter at POE (mm) | 5.1±1.4 | 5.3±2.1 | 0.343 |
| Tunnel length (mm) | 16.9±10.2 | 19.9±11.6 | 0.025 |
| Distance from POE to vessels (mm) | 3.0±1.8 | 2.5±1.6 | 0.001 |
| Distance from targets to pleural (mm) | 26.8±17.2 | 25.2±17.9 | 0.004 |
| mBTPNA: modified bronchoscopic transparenchymal nodule access; POE: point of entry | | | |
